# Anesthetic and analgesic techniques and perioperative inflammation may affect the timing of recurrence after complete resection for non-small-cell lung cancer

**DOI:** 10.3389/fsurg.2022.886241

**Published:** 2022-07-26

**Authors:** Katsuya Watanabe, Haruhiko Masuda, Daisuke Noma

**Affiliations:** ^1^ National Hospital Organization Yokohama Medical Center, Yokohama, Japan; ^2^ Department of Surgery, Yokohama City University, Yokohama, Japan; ^3^ General Thoracic Surgery, Saiseikai Yokohamashi Nanbu Hospital, Yokohama, Kanagawa, Japan

**Keywords:** non-small-cell lung cancer, hazard curve, recurrence, anesthetic technique, multimodal analgesia, inflammation-based score

## Abstract

**Introduction:**

It has been widely recognized that both surgery and anesthesia may increase the risk of cancer recurrence by inducing an inflammatory response and immunosuppression in various cancer operations. The present study explored using hazard curves how anesthetic and analgesic techniques regarding the host inflammation status affect the risk of recurrence over time in patients with non-small-cell lung cancer (NSCLC).

**Material and Methods:**

Clinicopathological data from patients who underwent complete pulmonary resection with pathological I–IIIB stage NSCLC from 2010 to 2020 were collected. The inflammation-based scores, including the C-reactive protein-to-albumin ratio (CAR), systemic immune-inflammation index (SII), Glasgow prognostic score (GPS), and modified GPS (mGPS), were calculated before surgery, and hazard curves indicating the changes in hazards over time were evaluated.

**Results:**

A total of 396 patients were eligible for the analysis. The median follow-up was 42.3 months. In total, 118 patients (29.8%) experienced recurrence, and 66.9% of those occurred within 24 months after surgery. According to the multivariate Cox regression analysis, volatile anesthesia (VA) (hazard ratio [HR], 1.69; 95% confidence interval [CI], 1.05–2.71), and elevated CAR (HR, 1.88; 95% CI, 1.18–2.99) were associated with a worse recurrence-free survival. The resulting hazard curve revealed that a delayed peak of recurrence was present in patients with a low CAR in the VA group and in those with intravenous flurbiprofen axetil administration in the propofol-based total intravenous anesthesia group (30 and 24 months after surgery, respectively).

**Discussion:**

Choosing anesthetic and analgesic techniques while taking inflammation-based scores into account may be useful for reducing the risk of and/or delaying recurrence in patients undergoing resection for NSCLC.

## Introduction

Lung cancer is a leading cause of cancer-related mortality worldwide. Surgery is a mainstay of treatment for early-stage non-small-cell lung cancer (NSCLC). However, early recurrence develops even after curative-intent surgery for patients with early disease due to clinically undetectable micrometastases (1).

The surgical insult itself can cause a sharp systemic inflammatory response and immunosuppression due to local tissue damage (2, 3). In the tumor microenvironment, it has been widely recognized that the inflammatory response substantially contributes to cancer development, invasion, and metastasis. Previous studies have reported that perioperative systemic inflammation and malnutrition are correlated with recurrence and a poor prognosis in various cancers (4, 5). In addition, inflammation-based scores, such as the C-reactive protein/albumin ratio (CAR), systemic immune-inflammation index (SII), Glasgow prognostic score (GPS), and modified GPS (mGPS), have been validated in lung cancer surgery (6–9).

Anesthesia has been reported to suppress cell-mediated immunity and increase angiogenesis and can, therefore, promote the proliferation and metastasis of cancer cells during the perioperative period, possibly influencing cancer recurrence and the prognosis for certain types of cancer (10). Of note, several metanalyses have shown that propofol-based total intravenous anesthesia (TIVA) is associated with a greater recurrence-free survival (RFS) and overall survival than volatile anesthesia (VA) across various cancer types (11, 12). However, at present, there is still insufficient evidence to recommend any particular anesthetic technique for patients undergoing cancer surgery. Hope remains that several ongoing large-scale prospective randomized studies will define the anti-cancer effect of anesthesia. However, few studies have focused on the timing of recurrence or evaluated the effect of anesthetic and analgesic techniques on the timing of recurrence.

Thus, in the present study, we examined hazard curves to evaluate how anesthetic and analgesic techniques in relation to inflammation-based scores affect the timing of recurrence after complete resection for NSCLC.

## Material and methods

### Setting

This retrospective cohort study was approved by the Institutional Review Board of National Hospital Organization Yokohama Medical Center (approval number 2021-21), which waived the requirement for obtaining informed consent and patient records.

### Study proportion

**Figure 1:**
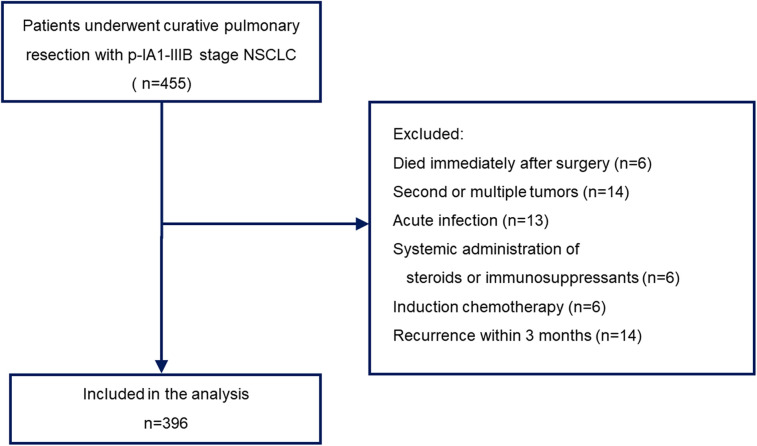
Flowchart detailing the selection of patients included in the study.

This study included patients who underwent complete pulmonary resection with pathological IA1 to IIIB stage disease between January 2010 and December 2020. Sublobar resection was allowed for patients who could not tolerate anatomic pulmonary resection or those with a peripheral ground-glass opacity (GGO)-dominant tumor. All other patients underwent lobectomy with lymph node dissection. Postsurgical pathologic tumor-node-metastasis (TNM) staging was performed according to the guidelines of the American Joint Cancer Committee (AJCC), 8th edition. The choice of anesthetic and analgesic techniques was made according to the anesthesiologist's preference. We obtained the following information from the electronic medical records: demographic data; blood cell count; blood biochemistry; height and weight; type of anesthetic used; use of epidural anesthesia; use of oral non-steroidal anti-inflammatory drugs (NSAIDs); use of intravenous NSAIDs; histological type of cancer; surgical procedure; pathological stage; adjuvant chemotherapy; and postoperative recurrence. Patients who died during or immediately after surgery, those with recurrence within 3 months, those with acute infection, those with active double cancer, those who received the systemic administration of steroids or immunosuppressants, and those who underwent preoperative chemotherapy were excluded. Finally, 396 patients with a single primary tumor were included in the analysis (Figure 1).

### Definition of inflammation-based scores

All inflammation-based scores were calculated from the preoperative routine laboratory data. The CAR was calculated from the serum C-reactive protein (CRP) value divided by the serum albumin value, and the SII was calculated as neutrophil × platelet/lymphocyte. The GPS was calculated based on the CRP and albumin values. Patients with elevated CRP levels (>1.0 mg/dl) and hypoalbuminemia (<3.5 g/dl) were considered as GPS 2, while those with CRP >1.0 mg/dl or albumin <3.5 g/dl were considered as GPS 1, and those with normal CRP and albumin levels were considered as GPS 0. Regarding modified GPS (mGPS), patients with elevated CRP (>0.3 mg/dl) and hypoalbuminemia (<3.5 g/dl) were assigned a score of 2, while those with only an elevated CRP level were assigned a score of 1, and those with a normal CRP level—regardless of the albumin level—were assigned a score of 0.

### Patient follow-up

Based on currently recommended follow-up guidelines (13, 14), a physical examination, chest radiography, and computed tomography (CT) findings of the chest and abdomen were evaluated. In patients with any signs of disease recurrence, CT of the chest and abdomen, brain magnetic resonance imaging (MRI), and fluorodeoxyglucose-positron emission tomography (PET) were performed as required. Recurrence was diagnosed based on the results of such diagnostic imaging studies and was confirmed by a pathological examination if needed.

### Statistical analyses

The results for continuous variables were expressed as the mean and standard deviation, while categorical variables were expressed as the frequencies and percentage. The RFS was defined as the time from surgery to recurrence or death. Patients without an event were censored at the time of final follow-up. The RFS was analyzed by both survival curves and hazard curves. Survival curves were expressed by the Kaplan–Meier plot, and differences in variables were analyzed by the log-rank test. To estimate hazard curves, we used the muhaz package [proposed by Muller and Wang (15)]. The cut-off values of the CAR and SII to test the predictive ability for disease recurrence were determined by a receiver operating characteristic (ROC) curve analysis. Multivariate Cox regression analyses were performed to calculate hazard ratios (HRs) to determine whether or not factors such as the age, sex, body mass index, anesthetic technique, use of epidural anesthesia, intravenous flurbiprofen axetil (FA), oral NSAIDs, the CAR, the SII, GPS, mGPS, surgical procedure, tumor histology, pathological stage, or adjuvant chemotherapy were potential independent predictors of recurrence.

All analyses were conducted using the R 3.6.2 (R Foundation for Computing, Vienna, Austria) software program. *P* values of less than 0.05 were considered to indicate statistical significance.

## Results

### Patient characteristics

The clinicopathological characteristics and treatment details of the 396 patients after curative-intent surgery for NSCLC are summarized in Table 1. The median age was 72 (range, 19 to 89) years old, and the median time to the first event was 42.3 (range, 1 to 134) months.

**Table 1:** Patient characteristics.

Variables	No. (%) or mean ± SD (range)
Age (years)	70.8 ± 9.3 (19–89)
Gender	
Male	245 (61.9)
Female	151 (38.1)
Body mass index (kg/m^2^)	22.6 ± 3.6
<18.5^a^	40 (10.1)
18.5–25.0^b^	266 (67.2)
≥25.0^c^	90 (22.7)
CAR	
<0.014	156 (39.4)
≥0.014	240 (60.6)
GPS	
0	339 (85.6)
1	35 (8.8)
2	22 (5.6)
mGPS	
0	307 (77.5)
1	62 (15.7)
2	27 (6.8)
SII	
<480	192 (48.5)
≥480	204 (51.5)
Surgical procedure	
Wedge resection	44 (11.1)
Segmentectomy	26 (6.6)
Lobectomy	312 (78.8)
Pneumonectomy	14 (3.5)
Anesthetic technique	
VA	296 (74.7)
TIVA	100 (25.3)
Epidural anesthesia	
Yes	281 (71.0)
No	115 (29.0)
Intravenous FA	
Yes	83 (21.0)
No	313 (79.0)
Oral NSAIDs	
Yes	372 (93.9)
No	24 (6.1)
Histological type	
Adenocarcinoma	275 (69.4)
Squamous cell carcinoma	90 (22.7)
Pleomorphic carcinoma	15 (3.8)
Adenosquamous carcinoma	10 (2.5)
Others	6 (1.6)
Pathological stage	
IA1	58 (14.6)
IA2	75 (18.9)
IA3	55 (13.9)
IB	58 (14.6)
IIA	10 (2.5)
IIB	71 (17.9)
IIIA	59 (14.9)
IIIB	10 (2.5)
Adjuvant chemotherapy	
Yes	113 (28.5)
No	283 (71.5)

^a^ Underweight.

^b^ Normal weight.

^c^ Overweight and obesity.

CAR, C-reactive protein/albumin ratio; FA, flurbiprofen axetil; GPS, Glasgow prognostic score; mGPS, modified GPS; NSAIDs, non-steroidal anti-inflammatory drugs; SII, systemic immune-inflammation index; SD, standard deviation; TIVA, total intravenous anesthesia; VA, volatile anesthesia.

The optimal thresholds for the CAR and SII based on ROC curve analyses were set at 0.014 and 480, respectively.

### Survival analysis findings

In total, 118 (29.8%) patients developed recurrence. The results of a multivariate Cox analysis of the RFS are given in Table 2. The anesthetic technique (*P* = 0.031), CAR (*P* = 0.008), and pathological stage (*P* < 0.001) were suggested as independent prognostic factors.

**Table 2:** Multivariate Cox analyses of RFS.

Variable	**Multivariate**		
	HR	95% CI	*P*-value
Age	1.002	0.9794–1.025	0.855
Male (ref: Female)	1.363	0.868–2.141	0.179
Body mass index ≥18.5, (ref: <18.5)	0.998	0.695–1.434	0.992
CAR ≥0.014, (ref: <0.014)	1.875	1.178–2.986	0.008
GPS 1 and 2 (ref: GPS 0)	1.122	0.652–1.933	0.677
mGPS 1 and 2 (ref: mGPS 0)	0.925	0.557–1.535	0.762
SII ≥ 480, (ref: <480)	1.270	0.850–1.899	0.244
Anesthetic technique, VA (ref: TIVA)	1.685	1.049–2.706	0.031
Epidural anesthesia (ref: no)	1.439	0.888–2.332	0.140
Intravenous FA administration (ref: no)	1.313	0.834–2.067	0.239
Oral NSAIDs (ref: no)	6.901	0.951–50.16	0.056
Histological type, non-Ad (ref: Ad)	1.254	0.807–1.950	0.314
Pathological Stage IIA or higher (ref: Stage I)	4.63	3.075–7.689	<0.001
Adjuvant chemotherapy (ref: no)	1.502	0.978–2.306	0.063

Ad, adenocarcinoma; CAR, C-reactive protein/albumin ratio; FA, flurbiprofen axetil; GPS, Glasgow prognostic score; mGPS, modified GPS; NSAIDs, non-steroidal anti-inflammatory drugs; RFS, recurrence-free survival; SII, systemic immune-inflammation index; TIVA, total intravenous anesthesia; VA, volatile anesthesia.

**Figure 2:**
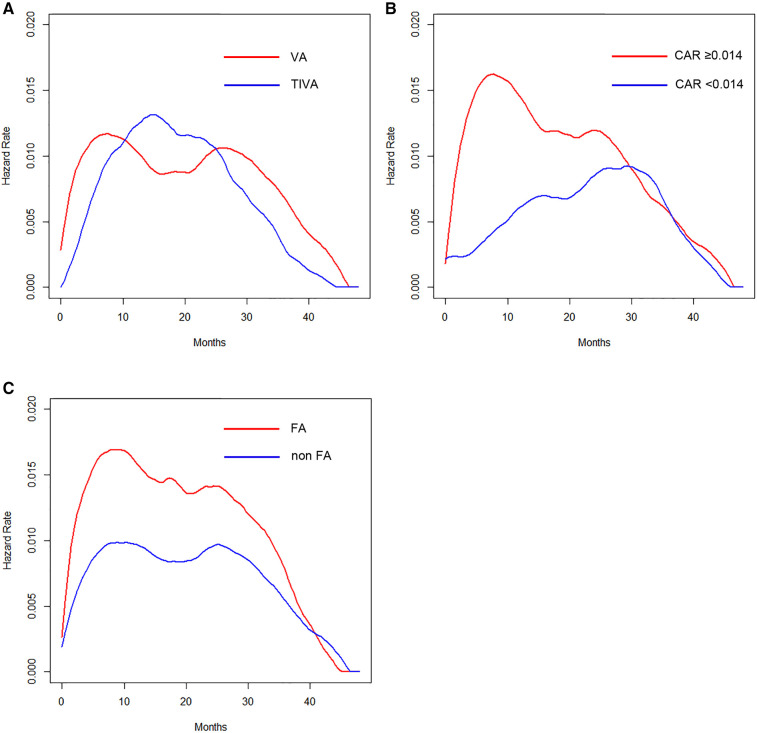
(**A**) Hazard curves according to anesthetic technique. (**B**) Hazard curves according to the CAR. (**C**) Hazard curves according to the perioperative intravenous FA administration.

The hazard of recurrence in patients with VA showed a bimodal pattern, whereas the hazard in those with TIVA displayed a single peak (Figure 2A). The hazard curve for patients with an elevated CAR value showed an earlier and higher peak (around 8 months) than in those with a low value (Figure 2B). Regarding the perioperative administration of NSAIDs, patients who received intravenous FA showed an early, large peak, whereas those without FA had several small humps (Figure 2C).

**Figure 3:**
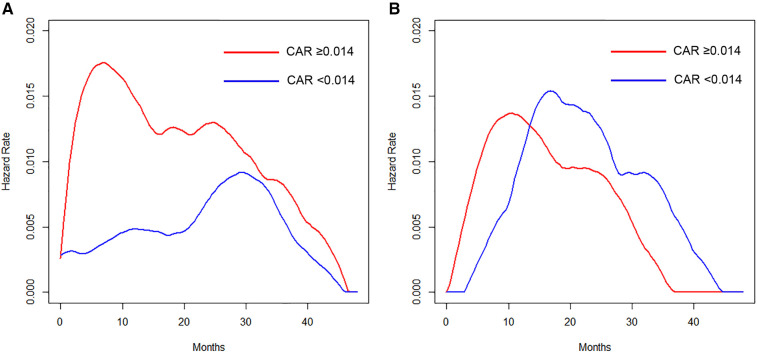
(**A**) Hazard curves of patients who received VA according to the CAR. (**B**) Hazard curves of patients who received TIVA according to the CAR.

The hazard curves of the anesthetic technique according to the CAR revealed that patients with a low CAR had a delayed and low peak of recurrence around 30 months, which was about 22 months later than in those with an elevated value in the VA group (Figure 3A). In contrast, the hazard and the timing of recurrence were similar between patients with elevated and low CAR values in the TIVA group (Figure 3B).

**Figure 4:**
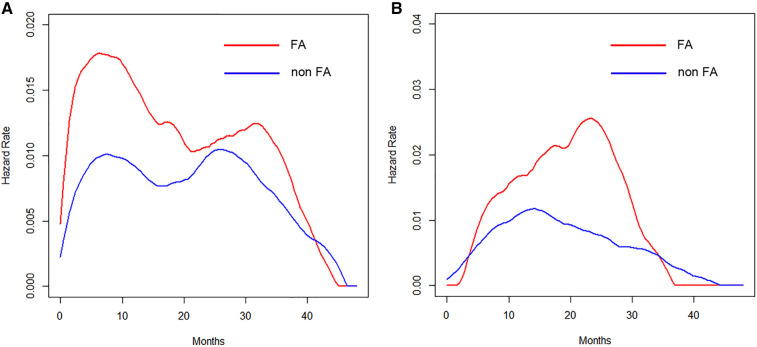
(**A**) Hazard curves of patients who received VA according to the perioperative intravenous FA administration. (**B**) Hazard curves of patients who received TIVA according to the perioperative intravenous FA administration.

The timing of recurrence for patients who received intravenous FA differed depending on the anesthetic techniques. An early peak at 8 months in the VA group (Figure 4A) and a delayed peak around 24 months in the TIVA group (Figure 4B) were observed.

## Discussion

The present findings revealed that the type of anesthesia, CAR, and pathological stage were independent predictors of the RFS. We also assessed the prognostic value of the anesthetic technique in relation to analgesic agents and the CAR in terms of estimating the timing of recurrence by hazard curves. The resulting hazard curves for patients who received VA showed a bimodal pattern, while the hazard curve for those who received TIVA showed a single peak pattern. Regarding the timing of recurrence in the VA group, patients with an elevated CAR value displayed an early, large peak (around 8 months), whereas the maximum peak was noted around 30 months for patients with a low CAR value, which was about 22 months later than in those with an elevated CAR value. Regarding the perioperative administration of NSAIDs in the TIVA group, patients who received intravenous FA showed an approximately 16-months-delayed but slightly larger peak around 24 months than the peak in those without intravenous FA.

Lung cancer often recurs even after complete resection and postoperative adjuvant chemotherapy. Clinically undetected micrometastasis and cancer cell dissemination during surgical manipulation are generally cited as major factors of local recurrence and/or distant metastasis. The recurrence risk for NSCLC changes throughout follow-up, with the peak reported to be around one year after surgery (16, 17). Systemic inflammation induced by surgery has been suggested to play a critical role in cancer growth, progression, and metastasis. In addition, anesthetic drugs in cancer operation can reportedly have an unfavorable effect on the immune system and affect patients' long-term prognosis. Such early recurrence after curative-intent surgery indicates that both surgery and anesthesia disrupt the homeostasis of the tumor microenvironment during the perioperative period. Numerous basic and clinical studies have suggested that the systemic inflammatory response may induce the production and secretion of inflammatory cytokines, prostaglandins, and vascular endothelial growth factor, inflammatory mediators that accelerate the proliferation of cancer cells, leading to disease progression in previously undetectable metastatic lesions (3, 4). However, these factors also regulate postoperative wound healing. The presence of malnutrition as well as systemic inflammation and immunosuppression in cancer patients is reportedly correlated with a poor prognosis in various malignancies. In addition, recent studies have suggested that inflammation-based scores, including the CAR, SII, GPS, and mGPS, may be useful prognostic indicators for patients with resected NSCLC (6–9). Furthermore, they are objective, simple, and cost-effective, preoperatively confirmed host-related factors that may reflect the systemic inflammation and nutritional status, unlike the tumor histological findings, postoperative TNM classification, and genetic biomarker values.

A number of previous studies have suggested that anesthetic techniques and analgesic agents may affect the postoperative recurrence of patients with various cancers. Although several retrospective studies reported no significant difference in the cancer-free or overall survival according to the type of anesthesia used (18). some metanalyses found that propofol-based TIVA was associated with a greater RFS and overall survival than VA (11, 12). In addition, propofol has been reported to be associated with a lower inflammatory response and an adverse immune response than VA during the perioperative period (19, 20). In order to prevent and delay recurrence, the results of the present study indicated that TIVA should, therefore, be actively recommended for patients undergoing curative-intent pulmonary resection with an elevated CAR value. However, at present, there is no clear evidence to recommend TIVA for the purpose of improving the prognosis over VA in patients undergoing cancer surgery, although no reports have found TIVA to be associated with a worse prognosis. Any firm conclusions will have to be drawn following the revelation of the results of a large prospective study currently in progress.

NSAIDs have various pharmacological actions, such as anti-inflammatory and analgesic effects. Some studies have reported that perioperative analgesic management using NSAIDs reduced the risk of recurrence after cancer surgery, suggesting that this may be attributable to the suppression of the systemic inflammation induced by surgery (21, 22). The detailed mechanisms behind the anti-cancer effect of perioperative administration of NSAIDs on patients undergoing surgery are unclear at present. We believe that the role of NSAIDs in surgery for NSCLC deserves further exploration through experimental studies, as such intervention as a part of multimodal analgesia is essentially low cost and trivial to implement; therefore, prospective clinical studies in large numbers of patients are warranted.

Cancer recurrence strongly affects a patient’s lifestyle and may affect their later life profoundly. Subsequent systemic treatment for recurrent NSCLC, including platinum-doublet chemotherapy, molecular-targeted drug, and immune checkpoint inhibitors, constitute a heavy physical and financial burden for patients who already have a reduced quality of life (QOL) following surgery. Considering these points, we feel it would be beneficial for patients if a particular anti-cancer treatment showed even a slight delay in the timing of recurrence after surgery compared with other treatments, despite no significant difference in the total risk of recurrence between those treatments. Our results indicated the possibility of perioperative management as a part of anti-cancer treatment for not only reducing the recurrence risk but also delaying the timing of recurrence. In performing curative-intent surgical resection, we have no control over host-related factors (e.g., sex, BMI, and inflammatory condition) or tumor-related factors (e.g., histopathology, tumor stage, and gene mutation); however, we can freely choose from a number of anesthetic and analgesic techniques, largely depending on the anesthesiologist’s preference, with the aim of minimizing pain and optimizing the recovery of the patient during the perioperative period. As the short perioperative period is disproportionately critical in influencing long-term cancer recurrence, in the future, we should broaden our vision to include the patients’ ultimate prognosis and select appropriate anesthetic techniques and analgesic drugs while ensuring perioperative patient safety. Perioperative management using safe, existing drugs may represent a cost-effective, novel alternative treatment option to standard anti-cancer treatment that maintains the QOL of patients if its effectiveness can be validated.

Several limitations associated with the present study warrant mention. First, it was a retrospective, single-institutional observational study. Therefore, there may have been some selection bias. In addition, we did not conduct propensity score matching in order to minimize the confounding in this study, as building a propensity score model that takes into account all of the relevant variables was difficult due to the small sample size. Furthermore, our analyses focused on heterogeneous populations through the various TNM stages of the disease. Despite the existence of these limitations, our study using hazard curves can provide some help in selecting appropriate anesthetic and analgesic techniques in lung cancer surgery.

## Conclusion

The anesthetic technique and CAR independently predicted the RFS for NSCLC. In addition, patients with a low CAR value in the VA group and those with perioperative intravenous FA administration in the TIVA group showed a delayed peak of recurrence. Perioperative management should be performed not only to optimize the recovery of the patient and minimize pain but also to select the most appropriate anesthetic technique and analgesics, taking into account the effect of inflammation status on the timing of recurrence in curative-intent surgery for NSCLC.

## Data Availability

The raw data supporting the conclusions of this article will be made available by the authors, without undue reservation.
